# Effects of the liaison nurse management on the infectious stroke complications: a randomized controlled trial

**DOI:** 10.1186/s12912-021-00802-0

**Published:** 2022-01-20

**Authors:** Zohreh Kalani, Sedigheh Ebrahimi, Hossein Fallahzadeh

**Affiliations:** 1 Medical Surgical Nursing Education, Department of Nursing, Research Center for Nursing and Midwifery Care, Shahid Sadoughi University of Medical Sciences, Yazd, Iran; 2 Critical Care Nursing, Department of Nursing, School of Nursing and Midwifery, Shahid Sadoughi University of Medical Sciences, Yazd, Iran; 3 Department of Biostatistics and Epidemiology, Research Center of Prevention and Epidemiology of Non-Communicable Disease, Shahid Sadoughi University of Medical Sciences, Yazd, Iran

**Keywords:** Nurse, Pneumonia, Stroke, Urinary tract infection

## Abstract

**Background:**

Two of the most serious complications after stroke are pneumonia, and urinary tract infection. Liaison nurse, from hospital admission to discharge and then at home helps patients with complicated caring issues stroke. This study investigates the effect of liaison nurse management on the incidence of pneumonia and urinary tract infection in patients with stroke after discharge from the hospital.

**Methods:**

This randomized controlled trial was conducted on 80 patients in a hospital in Iran. The intervention group was assessed and developed a caring program by the liaison nurse and the control group received routine care. Two weeks and two months after discharge, the patients were evaluated for the incidence of pneumonia and urinary tract infection. Collected data were analyzed using the Chi-square test. *P* < 0.05 was considered statistically significant.

**Results:**

The two groups were homogenous in terms of mean age; gender frequently distribution and having urinary catheter. The incidence of pneumonia in intervention and control groups (11.6% vs. 19.2%, *P* = 0.35) had no statistically significant differences, but there was a significant difference in the incidence of urinary tract infection (0% vs. 24.6%, *P* < 0.001).

**Conclusions:**

With liaison nurse performance, there was a significant difference in the incidence of urinary tract infection, in two months after discharge from hospital, but the incidence of pneumonia had no statistically significant differences in two groups. Nurse’s evaluation each patient individually according to needs, developing and monitoring the home-based care program, beyond overall education to these patients, could reduce some of complications of a stroke.

**Trial registration:**

This study is retrospectively registered by Iranian Registry of Clinical Trials with decree code: IRCT20170605034330N3 on April 4, 2018.

## Background

Stroke is the leading cause of functional impairments in adults which could affects both patients and their relatives in most regions. It is estimated to be a major health problem in the Middle East [1, 2], and in Iran is greater than in most western countries, with stroke occurring at younger ages [3].

According to World Health Organization (WHO) Stroke defined as rapidly developing clinical signs of focal or global disturbance of cerebral function, with symptoms lasting 24 h or longer or leading to death, with no apparent cause other than of [4]. Despite recent advances in emergency treatment of stroke and reduction of mortalities, it remains a major cause of mortality and disability [5]. The quality of care for stroke survivors is still lacking. Transferring hospital care home, especially for people with disabilities, such as stroke, is inefficient and is a stage of vulnerability that can be associated with a high risk of poor health outcomes [2]. Patients face significant barriers to recovery and an independent life, including cognitive and physical limitations, multiple medications, and a lack of social support [6].

Two thirds of patients experience at least one complication during the first week after stroke and 4 of 5 during three months of follow-up [7]. Medical complications are believed to be an important problem after acute stroke and present potential barriers to optimal recovery [8].

A major clinical complication that strokes survivors experience is infection [9]. Post-stroke complications have been reported at a rate of 30%that one-third include pneumonia and the other third urinary tract infections [10]. Both infections have relatively similar incidence but show very different disease processes [11].

Regarding the prevalence of complications in patients with stroke, comprehensive nursing care is highly needed for reducing the severity of complications, disability, and mortality within weeks after stroke [5]. Unfortunately, the gap between the hospital and patient support systems in the community leads to a decline in the quality of care and its continuation [12]. Improvement in quality of care, e.g., by a better preparation of the patient for discharge, is also an important aim in liaison nursing [13].

The discharge process from the hospital and his/her transfer to the home for the patient and the hospital is very important and should be performed gradually. Nurses can change treatment services for stroke survivors by improving the transition process [2]. Continuation of nursing interventions by the special stroke nurse as a liaison nurse after discharge has been beneficial for the patient and family through focusing on education and support [14]. Liaison nurse providing nursing services after discharge from the hospital, especially in chronic patients, will reduce readmission to hospital and thus continuity of care [15]. Liaison nurse, as a new role for nurses, has a positive effect on the quality of a patient’s outcome, from hospital admission to discharge and then at home [15]. Some health services in the world focus on this new role of the nurses adopted a terminology, such as “nurse liaison” or “discharge management nurse” [16].

Burton & Gibbon noticed a continuous nursing care system for discharged patients as “expanding the role of the stroke nurse”, and suggested establishing a therapeutic relationship between the nurse, the patient and the family may be crucial to fulfilling this aspect of the nurse’s role [14]. The liaison nurse in coordination with other members of the healthcare team ensure that all patients and their families are taken into consideration, nursing care and treatment are organized for each patient, and prevention of separate complications is performed [5].

The liaison nurse service was introduced in Australian hospitals to improve post-discharge care from the intensive care unit, reduce complications and hospitalization, and hospital mortality. The liaison nurse is expected to have enough knowledge and skills to continue patient care and communicate with other nurses and improve the patient’s outcomes [15]. The role of the liaison nurse is to improve the patient’s pre-discharge care plan, improve the relationship between hospital staff and healthcare staff in the community, and provide home care facilities with the help of hospital staff [17].

Liaison nurses through careful and comprehensive examination and the use of communication and technical skills can help with care in patients with multiple problems [18].. The findings of a systematic review in Iran stressed the role of the liaison nurse in improving the outcomes of patient care after discharge from the intensive care unit and suggested further research to provide more evidence on the concept of the liaison nurse [19]. In the only study found on liaison nursing for stroke patients, the researchers suggested the liaison nurses have been moderately successful in their jobs and therefore recommended future research on liaison nursing [13]. Despite the importance of the liaison nurse, this role is not defined for Iranian nurses and has no place in Iranian hospitals in a comprehensive and well-established way.

Ali Asghar Hospital in Shiraz is a teaching hospital that, like other teaching hospitals in Iran, operates under the management of the province’s University of Medical Sciences. According to the standardization guide for the stroke intensive care unit by the Secretariat of the Strategic Council, the Health Guides of the Deputy Minister of Health in 2020 https://treatment.sbmu.ac.ir/uploads/Standardization_of_Stroke_Care_Unit_ward.pdf, inpatients diagnosed with stroke receive care based on valid guidelines, but all care is usually limited to the patient’s hospitalization period.

This study is one of the first studies about liaison nursing in Iran and aimed to emphasize the liaison nurse as a role in the nursing. We have tried to show the role of a liaison nurse in the Iranian nursing system by investigate the effect of liaison nurse performance on the incidence of complications in patients with stroke after discharge from the hospital.

## Methods

### Study design and settings

This randomized clinical trial was performed in parallel in patients with stroke in March 2018 to February 2019. This study was done in neurology department of Ali-Asghar hospital in Shiraz, an urban area of Iran and after discharge, at patients’ home.

### Study population and sample

Patients were selected through convenience sampling on the first day of admission in the neurology department, based on inclusion and exclusion criteria. Inclusion criteria included age 45–85 years and a score of 16–25 based on the National Institutes of Health Stroke Scale (NIHSS), which strongly recommend for assessment of the stroke recovery [20]. The Cronbach’s alpha coefficients obtained for determining the internal consistency reliability of the Persian version of NIHSS were 0.893 for the entire group of patients [21]. Exclusion criteria were the presence of pneumonia, UTI and immobility before the onset of stroke. The risk of pressure sore based on Braden scale and the risk of falls by Morse scale were also determined.

At this point, before random division, written informed consent was signed by eligible patients who were willing to participate in the study. Afterward, the patients were assigned to the intervention or control group by simple random allocation with using random numbers table.

Based on reported incidence rate for UTI by Indredavik et al. [7] that was 16% during the first week and 27.9% for 3 months follow up;α = 0.05, *β* = 0.2; with a view to reducing the incidence of UTI by 20% (*p*_*1*_ = 21% and *p*_*2*_ = 1%); we used the following formula to calculate the sample size for comparing ratios in two independent groups. *n = (Z*_*α/2*_ *+ Z*_*β*_*)*^*2*^ *× [p*_*1*_ *(1-p*_*1*_*) + p*_*2*_ *(1-p*_*2*_*)]/ (p*_*1*_*-p*_*2*_*)*^*2*^*.*

Due to referring to patients’ homes in our country has many restrictions, because of obstacles related to cultural issues especially for female nurses, and especially if the patient resides in neighboring cities, and because in this study the liaison nurse only referred home for the intervention group, we inevitably used a ratio of the sample sizes (*r* = 1.3) for unequal sample sizes in groups [22]. The sample size was 35 in the interventional group and 45 in the control group.

**Fig. 1:**
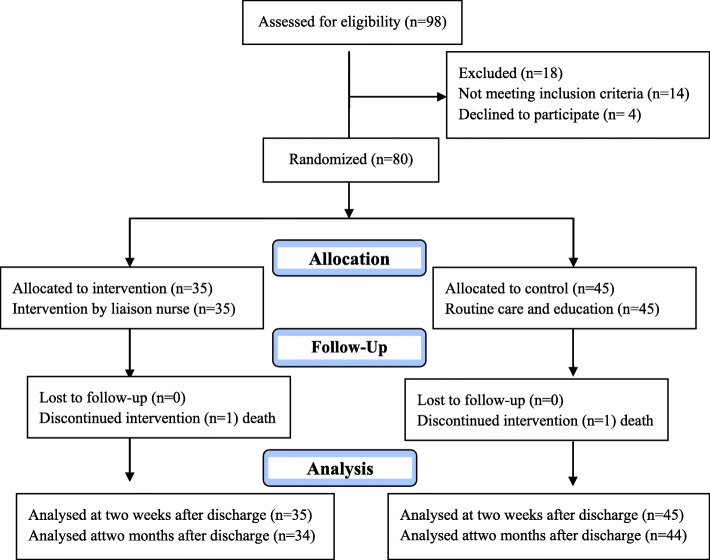
Flowchart of the study

Initially, 98 patients were enrolled into the studyon the first day of admission in the neurology department, 14 patients did not have the inclusion criteria, four subjects declined to participate. The study was performed on 80 allocated to the intervention and control groups. Two patients, one in each interventional and control group died during the study (Fig. 1).

Liaison nurses in this study were two registered nurses, a female that was a member of the research team who was studying intensive care nursing at the time of data collection, and a male nurse who was well-trained in the objectives of the study and how to intervene. Both liaison nurses had more than 10 years of experience in the neurology department of Ali Asghar Hospital.

### Intervention group (liaison nurse management)

Liaison nurse management consists of 4 phases, assessment, planning, Implementation, and evaluation.

#### Assessment phase

This phase of the management was performed when the patient was still hospitalized. Patients were visited individually by the liaison nurse to identify the probability of pneumonia and UTI based on risk factors of each such as dysphagia and having NGT, urinary status and having a catheter, paralysis status. Also, the potential and actual capabilities of the patient and his/her family were determined.

#### Planning phase

An individualized care plan was developed for each patient according to the priorities, objectives, and expected results, was determined.

#### Implementation phase

Patients received the nursing care according to individualized patient’s needs. Care was carried out individually by the researcher for each patient. One of the family members who were more empowered to care for the patient was received the necessary training for home care.

Training to the patient’s family on the patient’s bedside is done within 2 days of admission (the first day of oral education and the second day in practical form). The patient and his/her family were given a booklet containing guidelines for patient care in patients with stroke upon discharge from the hospital and was given a telephone number to communicate with the liaison nurse to resolve potential problems by coordinating the nurse.

After discharge from hospital, the liaison nurse visited the patient at home two sessions per week lasting 45–60 min for one month and provided necessary interventions aiming at resolving their caring issues.

Nursing care primarily focused on educating the patient and his family, including the basic concepts of stroke, the definition and nature of the disease, the types, causes, warning signs, and effects of stroke on the whole body.

Aimed at preventing complications, the necessary training was given to patients in the intervention group. In conscious patients, effective cough training, deep breathing, and encouragement to get out of bed as soon as possible were performed. It was noted that in patients with a high risk of falls, based on Morse scale, the patient’s activity should be performed under close supervision. In patients with low level of consciousness, special attention was paid to chest physiotherapy and discharge suction according to the needs of the patient. In order to prevent aspiration, based on the state of the swallowing reflex and the presence of dysphagia, the family and caregiver were taught on the patient’s bedside that how to feed the patient at home. To prevent aspiration, the patient should be placed in a semi-sitting position after feeding and using Chlorhexidine solution for oral hygiene.

Patients and their families were also instructed to contact the liaison nurse if there were any signs or symptoms of pneumonia, including fever, loss of consciousness, shortness of breath, and increased sputum.

In case of urinary incontinence, it was emphasized that a Foley catheter should not be inserted and diapers should be used to solve this problem. Perineal hygiene and diaper change at short intervals, especially in patients at high risk of pressure sore according to Braden scale, were taught. In case of urinary retention, it was instructed to use a temporary catheter at regular intervals instead of a Foley catheter. Permanent urinary catheter care training was given to the patient’s family, including keeping the urine bag clean and placing it below the level of the patient’s bladder, and washing the mea area.

Regular intake of fluids in patients with swallowing reflex orally and in case of dysphagia and swallowing problems through NGT or intravenous was emphasized.

Patients and their families were also instructed to contact the liaison nurse if there were any signs or symptoms of UTI including fever, decreased consciousness, and dysuria.

Care and instructions taken was based on guidelines and reliable sources [20, 23, 24]. After preparing the content of the intervention by the research team, it was reviewed by six members ofnursing department and after applying their opinions; it was presented in the project proposal. Since this study was a master’s thesis, along with the approval of the project proposal, the content of the intervention was reviewed and approved by the Graduate Committee of the faculty.

#### Evaluation phase

study group were followed up two weeks and two months after discharge for the incidence of pneumonia and UTI. In the event of symptoms that could cause a UTI or pneumonia, before two weeks and two months, the patients referred for visit the physician. The liaison nurse provided the necessary coordination to clinic and visits the neurologist.

#### Control group (standard care)

The control group patients were under routine hospital care. Prior to discharge, the patient and his/her family were given a telephone number to communicate with the liaison nurse to notify the liaison nurse of any signs and symptoms of pneumonia and UTI who were taught. Other than the above, the control group was evaluated for pneumonia and UTI, two weeks, and two months after discharge from the hospital.

### Occurrence of complications

In this study, the initial diagnosis for pneumonia based on the signs and symptoms included cough with sputum, dyspnea, fever and confusion and for UTI, fever, bad smell of urine, dysuria, and decreased consciousness level. Patients’ families in both groups were instructed to contact the liaison nurse if these symptoms occurred. In case of initial diagnosis by the liaison nurse, the patient was referred to the treating physician and the final diagnosis was made by the treating physician. However, it was up to the treating physician to confirm the nurse’s suspicions. Physician confirmation for pneumonia was based on signs and symptoms, results of blood tests, looking inflammation in the chest x-ray and positive result of sputum culture; and for UTI was based on signs and symptoms, results of blood tests and urine tests (urine analysis and urine culture). The blood tests were including a high white blood cell count, a high level of CRP and ESR more than 35.

### Data analysis

Data analysis was conducted by an expert statistician blinded to the study protocol. The collected data were analyzed by SPSS 20, using Chi-square and independent T tests. *P* < 0.05 was considered statistically significant.

## Results

In this study, 80 patients were examined, 35 patients in the intervention and 45 patients in the control group with mean ages of 64 ± 10 and 66 ± 08 years respectively (*P* = 0.37). Eighty percent of patients in the intervention group and 73.3% of patients in the control group had high school diploma or lower education.

Results of two months after discharge are for 78 patients (34 in intervention and 44 in control groups), because of dying two patients (one in each group) between two times of evaluation.

Considering the randomized distribution of the patients into the intervention and control groups, two groups had no statistically significant differences in terms of sex, age, comorbidities, paralysis, Risk of Pressure Sore based on Braden Scale, Risk of falls based on Morse Scale, having nasal-gastrointestinal tube (NGT), and urinary catheter (Table 1).

**Table 1:** The patients’ characteristics in the intervention and control groups

	Group		*X*^2^/*t/U*	*P* value
	Intervention (*n* = 35) N (%)	Control (*n* = 45) N (%)		
Age (years):				
≤ 65	18 (51.4)	19 (42.2)		
> 65	17 (48.6)	26 (57.8)	0.671	0.413*
Range	45–85	49–83		
mean ± SD	64.89 ± 10.19	66.98 ± 08.44	−1.003	0.319**
Sex:				
Male	19 (54.3)	23 (51.1)	0.080	0.778*
Female	16 (45.7)	22 (49.8)		
Comorbidities:				
Diabetes	17 (48.6)	22 (48.9)	0.001	0.978*
Hypertension	27 (77/1)	37 (82/2)	0.317	0.573*
Hyperlipidemia	18 (51/4)	21 (46.7)	0.179	0.673*
NIHSS:				
16–18	12 (34.3)	14 (31.1)		
19–22	14 (40.0)	18 40.0)	0.133	0.936*
23–25	9 (25.7)	13 (28.9)		
Range	16–25	16–25		
mean ± SD	20.40 ± 2.96	20.49 ± 3.05	−0.131	0.896**
Having paralysis:				
One side	28 (80.0)	35 (77.8)	0.062	0.969*
Two sides	2 (5.7)	3 (6.7)		
None	5 (14.3)	7 (15.6)		
Risk of pressure sore^a^:				
High	11(31.4)	16(35.6)	686.0	0.292***
Moderate	6(17.6)	13(28.9)		
Low	18(51.4)	16(35.6)		
Risk of falls^b^:				
High	18(51.4)	23(51.1)	777.0	0.911***
Moderate	10(6.28)	12(7.62)		
Low	7(20)	10(22.2)		
Having NGT:				
Yes	18 (51.4)	20 (44.4)	0.385	0.535*
No	17 (48.6)	25 (55.6)		
Having Urinary Catheter:				
Yes	21 (60.0)	27 (60.0)	0.000	1.000*
No	14 (40.0)	18 (40.0)		

*Chi-Square test, **t- test, ***Mann- Whitney test, ^a^Based on Braden Scale, ^b^Based on Morse Scale

As can be seen in Table 1, at beginning of the study, 18 patients (51.4%) in intervention group and 20 patients (44.4%) in control group had an NGT. Prescription of NGT by the treating physician was due to lack of gag reflex or dysphagia.

During visit two months after discharge, soft food was started for six patients (17.1%), Percutaneous Endoscopic Gastrostomy (PEG) was performed on four patients (11.4%) and eight patients (22.8%) still had NGT. In control group, during visit two months after discharge, soft food was started for five patients (11.2%), PEG was performed on four patients (8.9%) and11 patients (24.4%) still had NGT.

Two weeks and two months after discharge, in the intervention group, 5.7 and 5.9% of the patients, and in the control group 11.1 and 9.1% of the patients, suffered from pneumonia. There was no statistically significant difference between the two groups in the incidence of pneumonia two weeks (*P* = 0.45) and two months after discharge (*P* = 0.69) and in total between the groups (11.6 vs. 19.2, *P* = 0.35) (Table 2).

**Table 2:** Incidence of complications in the two groups after discharging from hospital

Complication		Intervention	Control	*P* value*
		(*n* = 35, 34)	(*n* = 45, 44)	
		N (%)	N (%)	
Pneumonia	After 2 weeks	2 (5.7)	5 (11.1)	0.397
	After 2 months	2 (5.9)	4 (9.1)	0.598
	Total	4 (11.6)	9 (19.2)	0.356
UTI	After 2 weeks	0 (0)	3 (6.7)	0.119
	After 2 months	0 (0)	7 (15.9)	0.015
	Total	0 (0)	10 (24.6)	0.0001

*Chi-square test

In the intervention group, 21 patients (60%) had a Foley catheter at the beginning of the study that of these, nine patients (25.7%) were due to urinary retention and 12patients (34.3%) were due to urinary incontinence. With the intervention of the liaison nurse at the end of the study, at the visit two months after discharge, only four patients (11.4%) had a Foley catheter. In control group,27patients (60.0%) had a Foley catheter at the beginning of the study that of these, 12 patients (26.7%) were due to urinary retention and 15patients (33.3%) were due to urinary incontinence. With the intervention of the liaison nurse at the end of the study, at the visit two months after discharge, 17 patients (37.8%) had a Foley catheter.

The incidence of UTIs two weeks and two months after discharge in the intervention group was 0%. Also, in the control group, it was 6.7 and 15.9%, respectively. The incidence of UTI in the two groups was not statistically significant two weeks (*P* = 0.25), but this difference was significant two months after the discharge (*P* = 0.01) and in total between the groups (*P* < 0.001) (Table 2).

## Discussion

To prevent post-stroke complications, the function of the liaison nurse can help reduce their problems. Stroke patients and their family face many problems after discharge, do not have normal life and are dependent on others. Nurses are in an ideal position to help in these situations, because they are constantly in clinical practice and are a mediator between the patient, family, and health care team [25]. The aims of liaison nursing are to improve the communications between hospital and home care and continuity and consequently improve the quality of care [13].

In this study, the effect of the liaison nurse performance on the prevention of the occurrence of infections stroke complications including pneumonia and UTI, was investigated.

Infection complications, most often pneumonia, contribute to increased mortality from stroke, prolong hospitalization, difficulties in care, reduce functional performance improvement and increased the cost of treatment [26].

The results of this study showed that the rate of pneumonia in liaison nurse group in two months was 11.6% less than in control group (19.2%). The incidence of complications from stroke is different in studies. It seems that the length of follow-up periods is effective in these differences. In a six-year period study of in-hospital stroke complications, reported the incidence of pneumonia to be 9% [27]. A multicentre study confirmed that at seven days after the acute stroke onset pneumonia occurred in 7.4% patients while during the first three months occurred in 13.6% of patients [28]. Other studies reported a range of incidence for pneumonia regardless to follow up period: 1–33% [26],10–20% [29], and 7–22% [30].

To prevent of aspiration pneumonia, the liaison nurse with practical education on how to feed the patient with a gastric tube, usually on the last day of hospitalization and during discharge, sought to increase the awareness of the family care givers. Care givers must be assessed for their capacity to provide the needed care and their readiness to assume the care giving role at home [2]. We found that involving the caregiver from the first day of admission prevent confusion and can provide better living conditions for the patient and his/her family. The results of this study showed that the presence of the liaison nurse in the patient’s bedside, education of the correct way and engaging the patient’s caregiver for suction, oral hygiene, mouthwash and tooth brushing that usually less considered after discharge, can reduce the incidence of pneumonia.

UTI, another commonly reported complication after stroke, has been shown to be associated with a poor outcome and mortality in the patient with stroke [31].

The results of this study showed that liaison nursing care resulted in a significant difference in the incidence of UTI between the groups.

The majority of UTIs in acute stroke are associated with the use of indwelling catheters; therefore, prolonged ones should be avoided. The risk of UTI is 3–10% per day of catheterization, approaching 100% after 30 days. Urinary incontinence and retention are common after stroke (29 to 58%), and limited mobility increases the likelihood of being catheterized [7]. The use of a Foley catheter for more than 48 hoursafter stroke increases the risk of urinary tract infection [20].

Despite the efforts of the liaison nurse for preventing ourpatients from having a permanent urinary catheter with a low level of consciousness, obtaining the consent of the physician for inserting a temporary catheter, and teaching how to insert it to the patient’s family, a limited number of family caregivers were willing to do the procedure. Although programs are designed to train family members in caring skills, they often lack the preparation and support to take on a caring role [2].

In this study, we obtained the better results in relation to the performance of liaison nurse in prevention of UTI. Although most patients had a permanent catheter, the incidence of UTI is 0% over the entire two month follow up period in the intervention group. In other studies, the incidence of UTI was reported to be 11% [31], 2–27% [26], 16–27% [7], and 7–28% [30], or even up to 43% [32].

Most studies have reported incidence of post-stroke complications, and few interventions have been conducted to prevent these complications. Some studies also examine the impact of community-based nursing interventions on other aspects of stroke patients’ lives. Burton and Gibon suggested the continued intervention of a stroke nurse after discharge is associated with improved patient perceptions of general health and reduced negative emotional reaction and perceived social isolation, reduced career strain, and reduced deterioration in physical dependence [14]. A randomized controlled trial of a nurse-led community-based self-management program for improving recovery among community-residing stroke survivors reported that 4 weeks program including home visit and follow-up phone calls improving self-efficacy, outcome expectation, and performance of stroke self-management behaviors [33]. In another study, tested phone-based intervention under nurse guidance after stroke is effective in improving blood pressure control and medication adherence among Ghanaian stroke patients within 1 month of symptom onset compared with standard of care [34].

In our study, once again, shown involving the family in the care of stroke patients has good results. Recommend that the family/caregiver of the stroke patient, as essential members of the rehabilitation team, should be informed and involved in decision making and treatment planning as early as possible, and throughout the duration of the rehabilitation process [20].

To the best of the authors’ knowledge, this is the first randomized controlled trial which examines the liaison nurse performance among Iranian community-residing stroke survivors.

Since this project was a student thesis, we were faced with a time and budget limit. It seems that to determine the effect of interface liaison nurse performance in stroke patients, designing and conducting long-term and large-scale studies may produce different results and more persuasive evidence. In this study the effect of the intervention may be overestimated due to the small sample size. The other limitations of this study were convenience sampling and also because of the nature of the study, it was not possible for patients to be blinded to being in the intervention or control group.

Education and most importantly, not leaving the patient alone after the discharge, continuing care and monitoring of the patient at home are interventions by the liaison nurse.

## Conclusion

The results of this study showed that with liaison nurse performance, there was a significant difference in the incidence of UTI, in stroke patients in two months after discharge from hospital, but the incidence of pneumonia had no statistically significant differences in two groups. This study showed the nurse’s evaluation each patient individually according to her/his needs, developing and monitoring the home-based care program, beyond overall education to these patients, could reduce the complications of a stroke.

## Data Availability

The datasets used and/or analyzed during the current study are available from the corresponding author on reasonable request.

## Abbreviations

CRP: C-Reactive Protein
DM: Diabetes Melitus
ESR: Erythrocyte Sedimentation Rate
HDL: Hyperlipidemia
HTN: Hypertension
NGT: Nasal Gastric Tube
NIHSS: National Institutes of Health Stroke Scale
PEG: Percutaneous Endoscopic Gastrostomy
UTI: Urinary Tract Infection
WHO: World Health Organization.
